# Improving outcomes for the critically ill in developing countries: what is next?

**DOI:** 10.5935/0103-507X.20150054

**Published:** 2015

**Authors:** Rahul Kashyap, Manuel Hache-Marliere, Srdjan Gavrilovic, Ognjen Gajic

**Affiliations:** 1 Checklist for Early Recognition and Treatment of Acute Illness and Injury (CERTAIN) team.; 2 Anesthesia and Critical Care Medicine, Mayo Clinic - Rochester, Minnesota, United States.; 3 United States Critical Injury and Injury Trial (USCIIT) Group.; 4 CEDIMAT - Santo Domingo, Dominican Republic.; 5 The Institute for Pulmonary Diseases of Vojvodina, Sremska Kamenica, Serbia.; 6 Faculty of Medicine, University of Novi Sad - Novi Sad, Serbia.; 7 Pulmonary and Critical Care Medicine, Mayo Clinic - Rochester, Minnesota, United States.

## Critical care in developing countries

Critical care is a complicated, high risk, resource-dependent environment. Developing countries face common barriers to delivering quality emergent care due to the lack of supplies, coordination, infrastructure, technology, and human resources (e.g., competency-based education, multi-disciplinary staff and access to the most recent literature).^(1)^ More importantly, the capacity to provide care for critically ill patients in intensive care units (ICUs) of low-income countries is unknown. Most developing countries lack published data on ICU capacity.^(2)^ Importantly, a previous observational study aimed to assess the worldwide burden of critical illness, but only an insignificant sample (2 of 730 centers) of ICUs in low-income countries was taken into account.^(3)^

Poor access to material resources and skilled personal pose a significant barrier to care improvement. Indeed, severity-of-illness-adjusted mortality is inversely related to gross national income.^(3)^ Nevertheless, a recent survey performed by the CERTAIN (Checklist for Early Recognition and Treatment of Acute Illness and INjury; www.icertain.org) Investigators network in 15 ICUs from 11 low- and middle-income countries showed that 77% of ICUs singled out lack of protocols and trained staff, which are the most important barriers to improving the care and outcomes of critically ill patients as opposed to cost-dependent variables, such as equipment or supplies.^(1)^ Basic resources, such as standardized supportive care and trained personnel have been cited as instrumental in changing the outcomes for catastrophic/challenging diseases, such as the Ebola virus disease in resource-limited settings.^(4)^

## Outcome research

The first step in solving the problem is to measure the problem, i.e., collect data for processes of care and patient outcome measures. Out of the various critical care syndromes, sepsis heavily afflicts morbidity and mortality in ICUs^(1,3)^ regardless of the resources at hand. Thus, focused efforts to understand sepsis outcomes and interventional studies are essential in unlocking the keys to the success of critical care in developing countries. Developing countries in Asia and Africa report mortalities as high as 45% for sepsis.^(5,6)^ The common denominators for such high mortality rates are low adherence to protocols^(6)^ and the lack of adaptive-innovative quality improvement strategies.^(7)^

Nevertheless, low- to non-cost interventions, such as early sepsis management provided by a dedicated study medical officer was shown to substantially reduce the 30-day mortality rate for sepsis patients.^(5)^ Additionally, in a national study in Brazil, implementation of a multifaceted sepsis education program increased compliance with the entire sepsis bundle by four-fold (from 13% to 62%), resulting in patients being identified progressively earlier and at a lower illness severity, and a decrease in hospital mortality and costs.^(8)^

## Checklists in critical care

Intensive care units heavily rely on continuous electronic monitoring, frequent blood tests and imaging modalities. This surplus of complex information may overwhelm clinicians and impair decision-making. As previously described, establishing a systematic approach improves outcomes without consuming means. A structured approach to the management of sepsis or ventilatory failure (i.e., care bundles) has become nearly universal in the ICUs of high-income countries.

The use of checklists is an effective strategy that ensures timely error-free compliance with care bundles. Checklists have emerged from the nuclear and aviation industry into high-risk hospital settings, such as trauma resuscitation and surgical suits. In the ICU, checklists have improved the processes of care, patient safety and morbidity.^(9)^ However, despite clear benefits and wide appeal, only 38% of ICUs in low-middle income countries report the use of some checklists during daily rounds upon admission. Only 15% of ICUs have reported the use of any type of checklists for acute resuscitation.^(1)^ When used in trauma resuscitation in a randomized, controlled interventional study, the computer-assisted decision support (checklist) decreased the number of errors and improved protocol compliance.^(10)^

The CERTAIN initiative is a multidisciplinary international quality improvement effort, which uses cloud-based electronic checklist and decision support tools to facilitate the best practices during admission/resuscitation and daily rounds of critically ill patients. CERTAIN methodologies and algorithms are applicable to a wide variety of pre-hospital, austere setting, and transport scenarios. Current and future development of CERTAIN methodologies and algorithms are important in the care of patients with life-threatening physiological conditions who manifest ongoing surveillance and resuscitation requirements.

## Remote education

Importantly, mere physical space does not make an ICU education and retaining skills are crucial to effectively care for critically ill patients. Nearly one-third of low-middle income countries are not staffed by specialists in critical care, and only approximately half of these countries had access to medical journals (54%) and continuing health education (61%).^(1)^ This number is even lower, in some parts of the world. More importantly, access to ICU care is limited. Most critically ill patients are treated outside of the ICU during the early, golden hours of critical illness when error-free care is the most important but is the least likely to occur. To overcome this barrier, there is a need for low cost and easily available training resources.

In 2011, over 135 web-based education resources for critical care were identified, including tutorials, self-directed learning modules, interactive case studies, webcasts, podcasts, and video-enhanced programs.^(11)^ Even practice-based skills, such as procedural ultrasound can be acquired effectively via remote education.^(12,13)^ Web-based education can convey the same results as classroom instruction with greater time flexibility,^(13)^ and, in some cases, may even lead to greater proficiency.^(12)^

Although there are a wide variety of resources, critical care remote education is still in its infancy. Education and research needs to be built around the needs of developing countries. These countries have limited access to resources, and thus, there is a need to design innovative approaches to bypass their constricted states and reach a wider audience. Currently, the CERTAIN group investigators are testing the delivery best-care practice on critical care patients via a novel, web-based, simple, electronic decision support tool with remote education, use of checklists and two-way video remote simulation assessment.^(14)^ Advances in telemedicine (e-ICU) are likely to complement remote education and further enhance knowledge delivery to the patient bedside anywhere.^(15)^

## Conclusion

Critical care clinicians face a myriad of challenges while delivering quality care. Research endeavors in developing countries require strategic, low-cost solutions. The use of checklists, bundles and structured processes have been instrumental in decreasing the number of errors of omission and complications in critically ill patients (Table 1).

**Table 1:** Checklist and remote education for outcome improvement

How checklists and remote education improve outcomes for critically ill patients in developing countries	
•	Increase adherence to protocols
•	Decrease errors of omission
•	Standardize care despite resource constrictions
•	Enhance knowledge base
•	Streamline the workflow
•	Improve outcome and decrease cost

Multi-national quality improvement projects should focus on high-risk conditions for which simple, timely, error-free interventions can make the most difference (e.g., early recognition of sepsis, shock and respiratory failure; adequate resuscitation; appropriate sedation and ventilation policies, and, when appropriate, palliative care). Furthermore, there is a need for translating research outcomes into sustainable education platforms. The CERTAIN initiative, as a multidisciplinary international effort, has the potential to create a model for future investigations for 'how to' implement novel knowledge translation interventions in resource-poor settings for better care and lower cost.
